# MBD3 promotes epithelial-mesenchymal transition in gastric cancer cells by upregulating ACTG1 via the PI3K/AKT pathway

**DOI:** 10.1186/s12575-023-00228-9

**Published:** 2024-01-05

**Authors:** Huizhi Wang, Jingyu Min, Yuntao Ding, Zhengyue Yu, Yujing Zhou, Shunyu Wang, Aihua Gong, Min Xu

**Affiliations:** 1 Department of Gastroenterology, Affiliated Hospital of Jiangsu University, Jiangsu University, 438 Jiefang Road, Zhenjiang, 212001 China; 2 Department of Gastroenterology, Changshu No.2 People’s Hospital, 68 Haiyu South Road, Changshu, 215500 China; 3 Department of Cell Biology, School of Medicine, Jiangsu University, 301 Xuefu Road, Zhenjiang, 212013 China

**Keywords:** MBD3, EMT, ACTG1, PI3K/AKT, Gastric cancer

## Abstract

**Background:**

Gastric cancer (GC) is a common malignancy and a leading cause of cancer-related death with high morbidity and mortality. Methyl-CpG binding domain protein 3 (MBD3), a key epigenetic regulator, is abnormally expressed in several cancers, participating in progression and metastasis. However, the role of MBD3 in GC remains unknown.

**Methods:**

MBD3 expression was assessed via public databases and validated by western blotting and quantitative real-time polymerase chain reaction (qRT-PCR). The prognosis of MBD3 was analysed via bioinformatics based on the TCGA dataset. The migration, invasion and proliferation of GC cells were examined by transwell, wound healing, cell counting kit (CCK)-8, colony-formation and xenograft mouse models. Epithelial-mesenchymal transition (EMT) and phosphatidylinositide 3-kinases/ protein Kinase B (PI3K/AKT) pathway markers were evaluated by Western blotting. RNA sequencing was used to identify the target of MBD3.

**Results:**

MBD3 expression was higher in GC tissues and cells than in normal tissues and cells. Additionally, high MBD3 levels were associated with poor prognosis in GC patients. Subsequently, we proved that MBD3 enhanced the migration, invasion and proliferation abilities of GC cells. Moreover, western blot results showed that MBD3 promoted EMT and activated the PI3K/AKT pathway. RNA sequencing analysis showed that MBD3 may increase actin γ1 (ACTG1) expression to promote migration and proliferation in GC cells.

**Conclusion:**

MBD3 promoted migration, invasion, proliferation and EMT by upregulating ACTG1 via PI3K/AKT signaling activation in GC cells and may be a potential diagnostic and prognostic target.

**Supplementary Information:**

The online version contains supplementary material available at 10.1186/s12575-023-00228-9.

## Introduction

Gastric cancer (GC) is a lethal and aggressive malignancy with high morbidity and mortality [1]. Due to the lack of early detection, the prognosis of GC is poor and the survival rate of patients is low [1]. Although modern medical technology has made great progress, its efficacy in the treatment of GC is limited [2]. The identification of novel biomolecules may provide potential therapeutic strategies for GC. Therefore, it is necessary to identify potential molecular mechanisms in GC progression.

Increasing attention has been given to epigenetic regulation in the development of GC, and the methyl-CpG binding domain (MBD) protein family plays an important role in epigenetic regulation [3, 4]. MBD3 is a key component of the nucleosome remodeling and deacetylase (NuRD) complex, binding methylated DNA to regulate genes with these modifications on the promoter [5]. MBD3 was previously found to play a key role in embryonic stem cell pluripotency and can also participate in the process of cancers [6, 7]. Studies have found that MBD3 plays different roles in different types of cancer. In hepatocellular carcinoma [8], MBD3 promotes metastasis by inhibiting the tumour suppressor tissue factor pathway inhibitor 2, while MBD3 inhibited epithelial-mesenchymal transition (EMT) via TGF-β/Smad signalling in pancreatic cancer cells [9]. However, the role of MBD3 in GC remains unclear.

EMT has been widely recognized as one of the key molecular steps in tumor cell invasion and metastasis to distant organs [10]. Aberrant activation of EMT is associated with malignant properties of tumor cells, including promoted migration and invasiveness. In the EMT process, GC cells acquire mesenchymal properties, express the mesenchymal markers N-cadherin and Vimentin, and lose cell–cell junctions and epithelial marker E-cadherin expression [11]. Additionally, the occurrence of EMT is related to many factors, such as epigenetic modification, transcription factors, microRNAs and long non-coding RNAs [12]. Therefore, we explored the effect of MBD3 on migration, invasion, proliferation and EMT in GC cells.

In this study, we aimed to explored the role of MBD3 in GC and prove that MBD3 may serve as a candidate target for GC diagnosis and prognosis.

## Materials and methods

### Data source

The RNA sequencing and clinical data (survival time and survival status) of patients with GC and other tumors were collected from the Genotype Tissue Expression Project (GTEx) database and the Cancer Genome Atlas (TCGA) database.

### Pathological sample collection

A total of 3 samples of gastric cancer tissues and their matched paracancerous tissues were collected between March 2023 and April 2023 at the Affiliated Hospital of Jiangsu University. This study was approved by the medical ethics committees of the Affiliated Hospital of Jiangsu University and was conducted in line with the Declaration of Helsinki.

### Immunohistochemistry (IHC)

Tumor tissues and paracancerous tissues were fixed in 10% formalin, paraffin-embedded, sliced into 4 ~ 6 μm sections, and placed onto slides. After deparaffinization, rehydration and microwave antigen retrieval, the slides were incubated with MBD3 (Proteintech, Cat No. 14258–1-AP) antibody at a 1:800 dilution at 4 °C overnight. Then, the slides were incubated with secondary antibody at room temperature for 30 min and stained with DAB substrate, followed by hematoxylin counterstaining.

### Cell culture

The human gastric normal epithelial cell line GES-1 and GC cell lines (SGC-7901, MGC-803 and HGC-27) were stored by the Institute of Medical Science, Jiangsu University (Zhenjiang, Jiangsu, China). All cells were tested and authenticated by short tandem repeat analysis. Cells were cultured in DMEM (Meilunbio, Dalian, China) containing 10% fetal bovine serum, 1% penicillin and streptomycin (HyClone, South Logan, UT, USA) in a humidified incubator at 37 °C and 5% CO_2_.

### Western blotting

Cells were rinsed with cold PBS, and then lysed with RIPA buffer containing 1% PMSF, 1% protease inhibitor, 5% 2-mercaptoethanol and 93% 2 × loading buffer. The cell lysate boiled at 100 °C for 10 min was centrifuged at 12 000 × g for 10 min. Subsequently, the total protein was separated by SDS-PAGE and transferred onto PVDF membranes for immunoblot assays. Membranes were blocked with 5% BSA for 1 h at room temperature and incubated with primary antibodies overnight at 4 °C. The membranes were washed, incubated with the respective HRP-conjugated secondary antibody for 1 h at room temperature, and then visualized with an ECL detection system (Amersham Pharmacia Biotech, Little Chalfont, UK). The antibodies were: rabbit anti-MBD3 (Proteintech, Cat No.14258–1-AP), rabbit anti-β-Tubulin (Abcam, CAT 21058), rabbit anti-MMP2 (ImmunoWay, CAT YT2798), rabbit anti-MMP9 (ImmunoWay, CAT YT1892), rabbit anti-Flag (ABclonal, CAT AE063), rabbit anti-Snail (Cell Signaling, CAT 3879), rabbit anti-α-SMA (Cell Signaling, CAT 68463), rabbit anti-Vimentin (Cell Signaling, CAT 5741), rabbit anti-N-cadherin (Cell Signaling, CAT 13116), rabbit anti-β-catenin (Cell Signaling, CAT 8480), rabbit anti-E-cadherin (Cell Signaling, CAT 3195), rabbit anti-p-PI3K (Sigma, CAT SAB4504314), rabbit anti-PI3K (Santa Cruz, CAT sc-1637), rabbit anti-p-AKT (Santa Cruz, CAT sc-271966), rabbit anti-AKT (Santa Cruz, CAT sc-5298), rabbit anti-p-mTOR (Santa Cruz, CAT sc-293133), rabbit anti-mTOR (Santa Cruz, CAT sc-517464), rabbit anti-ACTG1 (Affinity, CAT AF0115).

### Quantitative real-time polymerase chain reaction (qRT-PCR)

RNAiso Plus (Invitrogen) was used to extract total RNA according to the manufacturer’s protocol. Complementary DNAs (cDNAs) were synthesized from RNA samples (1 μg) by RevertAid First Strand cDNA Synthesis Kit (Thermo Scientific). qRT-PCR was performed via a SYBR Green Mix kit (Bio Rad Laboratories, Hercules, CA). Analysis of the relative expression was based on the 2^−ΔΔCt^ method. The primers were as follows: GAPDH-forward: 5'-GGTGAAGGTCGGTGTGAACG-3' and GAPDH-reverse: 5'-CTCGCTCCTGGAAGATGGTG-3'; MBD3-forward: 5'-CGGCCACAGGGATGTCTTTT-3' and MBD3-reverse: 5'-TGCTGGGGTGGTTGGTAATC-3'; MMP2-forward: 5'-CACAGGAGG AGAAGGCTGTG-3' and MMP2-reverse: 5'-GAGCTTGGGAAAGCCAGGAT-3'; MMP9-forward: 5'-TTCAGGGAGACGCCCATTTC-3' and MMP9-reverse: 5'-TGTAGAGTCTCTCGCTGGGG-3'.

### Prognostic analysis

Kaplan–Meier plots were used to assess the relationship between MBD3 expression and the prognosis (OS, Overall survival, DSS, Disease-specific survival) of cancers. The area under the curve (AUC) curves were generated by analyzing the data using the timeROC package and the results were visualized by ggplot2. Risk score maps were visualized with the ggplot2 package. The survival package was used for proportional hazards hypothesis testing and Cox regression analysis. The rms package was used for calibration analysis and visualization. The survival package was used for proportional hazards hypothesis testing and Cox regression analysis, and the rms package was used to construct and visualize the nomogram correlation model. The forest map was visualized by ggplot2. The proportional hazards hypothesis test and fitted survival regression were performed using the survival package, and the results were visualized using the survminer package and the ggplot2 package. Hypothesis testing was performed using the log-rank test, and *P* < 0.05 was considered statistically significant.

### Cell transfection

The plasmids sh-EGFP, sh-MBD3, 3 × Flag-vector (shown as Vector) and Flag-MBD3 were obtained from the Institute of Medical Science, Jiangsu University (Zhenjiang, Jiangsu, China). The sequence was confirmed through DNA sequencing. Lipofectamine 2000 reagent (Invitrogen, Carlsbad, CA, USA) was used for cell transfection based on the manufacturer’s protocols. Each well of a 6-well plate contained 2 μg plasmids and 10 μL Lipofectamine 2000.

### Cell migration and invasion assay

Transwell assays were performed in a Transwell chamber (pore diameter, 8 μm; MilliporeSigma). Transfected GC cells were resuspended in serum-free medium, and subsequently seeded into the upper layer of the chamber (6 × 10^4^ cells/100 μl) while complete medium was added to the chamber bottom. After 20 h, the cells on the lower surface were fixed with 4% polyformaldehyde (Aladdin, Shanghai, China) and stained with 0.05% crystal violet (Beyotime, Shanghai, China) for 30 min at room temperature whereas the cells on the upper surface were wiped slightly. An inverted light microscope (Olympus Corporation) was used to capture the images. The cell invasion assay was similar to the migration assay except that cells were seeded in Matrigel-coated Transwell inserts (BD Bioscience, Corning, NY, USA).

### Wound healing assay

Transfected GC cells (2 × 10^5^/well) were seeded in 24-well plates for 24 h and then scratched in wells with a 10 μl micropipette tip. Nonadherent cells were washed away with phosphate-buffered saline (PBS), and fresh medium was added. Images of wounds were acquired at 0 h and 20 h with a microscope. The wound-healing rate was calculated as follows: 100% × [(wound width at 0 h − width at 20 h)/width at 0 h].

### Cell Counting Kit (CCK)-8 assay

After 48 h of transfection, 1 × 10^3^ cells/100 μL were seeded into 96-well plates in each well. After 12 h, the cells adhered to the wall. Then, the cells were incubated with 100 μl CCK8 reagent mixture (10 μl CCK8 reagent: 90 μl DMEM) without light for 2 h at 37 °C. The results were measured at 450 nm absorbance by a microplate reader (Bio-Rad, Hercules, CA, USA) and analyzed via GraphPad Prism version 8.

### Colony formation assay

Single-cell suspensions were plated in 6-well plates at 1 × 10^3^ cells/well and maintained at 37 °C and 5% CO_2_ for 10 ~ 14 days. The supernatant was changed every three days. Finally, 4% paraformaldehyde was used to fix colonies and 0.5% crystal violet was used for staining for 30 min. The number of visible colonies was counted by ImageJ.

### Xenograft mouse model

The protocol was approved by the Ethics Committee of Jiangsu University. MGC-803 cells (2 × 10^6^ cells/site) stably transfected with vector or Flag-MBD3 were subcutaneously injected into 5-week-old BALB/c nude mice (Shanghai SLAC Laboratory Animal Co., Ltd., Shanghai, China) to generate xenografts. There were five female mice in each group. Tumor volume was measured weekly after injection and calculated using the formula: length × width × height × π/6 (the tumor is ellipsoid).

### RNA sequencing

MGC-823 cells (3 × 10^5^) were seeded into a 6-well culture plate and transfected with plasmids for 48 h. Cells were washed with cold PBS twice, and total RNA was isolated using RNAiso Plus (Takara) following the manufacturer's protocol. After verifying its concentration and integrity, the qualified RNA samples were subjected to PCR amplification to construct a cDNA library. Cluster generation and sequencing were performed on a NovaSeq 6000 S4 platform using a NovaSeq 6000 S4 Reagent kit V1.5. To guarantee the data quality that was used for analysis, the useful Perl script was used to filter the original data to remove low-quality sequences. The reference genomes and the annotation file were downloaded from the ENSEMBL database (http://www.ensembl.org/index.html).

### Statistical analysis

Each experiment was performed separately at least three times. The data are presented as the mean ± standard deviation (SD). Student’s t-test determined the statistical significance between two groups, while two-way analysis of variance (ANOVA) was used to analyze data from more than 3 groups. The results were analyzed via GraphPad Prism 8 software. *P* < 0.05 was considered statistically significant.

## Results

### MBD3 is abnormally expressed in GC tissues and cells

**Fig. 1:**
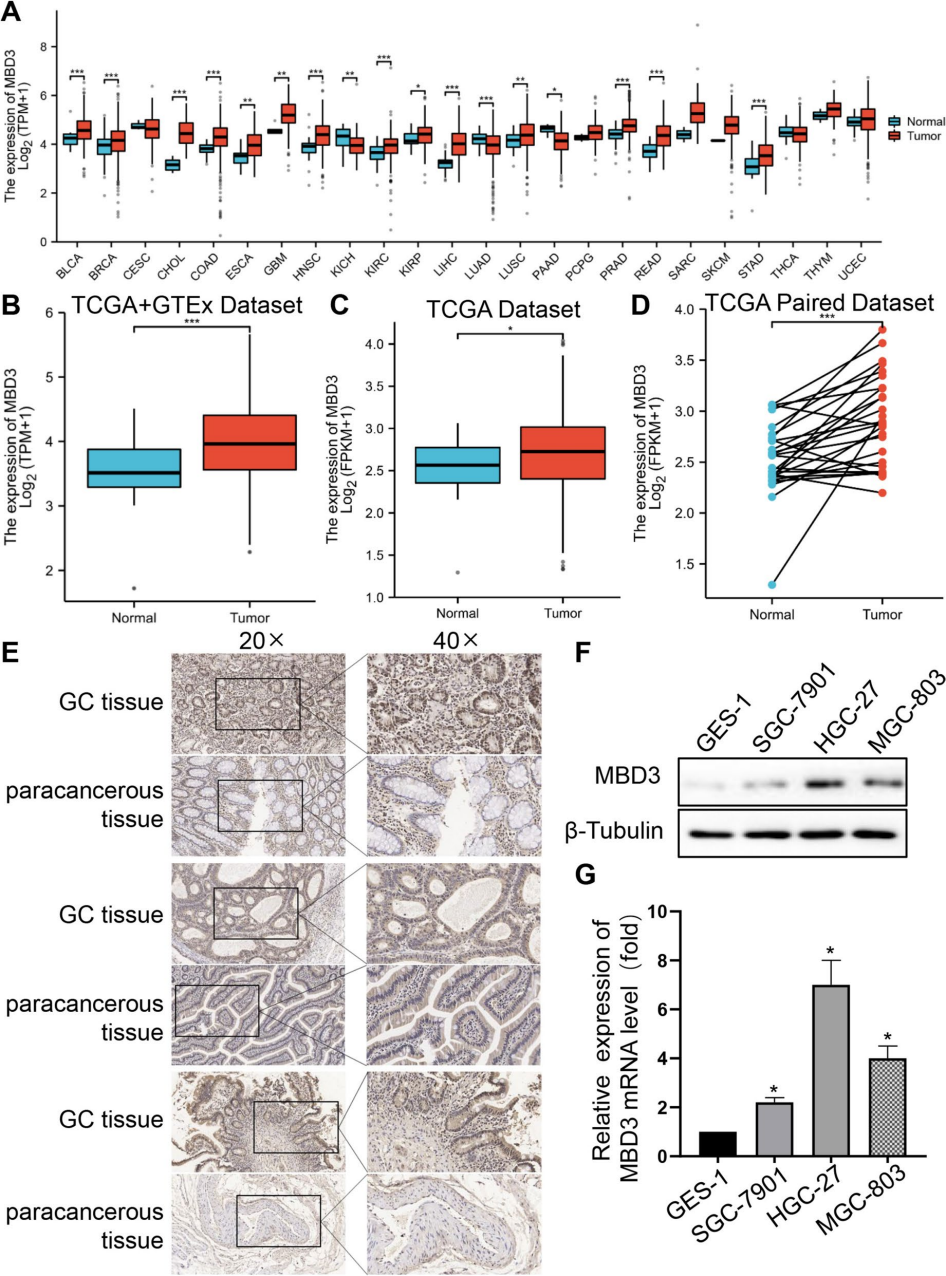
The expression of MBD3 in GC tissues and cells. **A** MBD3 expression in different cancer types based on the TCGA database. **B** The expression of MBD3 was higher in GC than in normal gastric tissues from the GTEx database (normal 174 patients) and (**C**) TCGA database (normal 36 patients, tumor 414 patients). **D** MBD3 expression in TCGA-paired GC and normal tissues (normal 27 patients, tumor 27 patients). **E** IHC staining for MBD3 in GC and matched paracancerous tissues from three representative patients. Original magnifications 20 × and 40 × (inset panels). **F**, **G** Western blotting and qRT-PCR were used to measure the relative expression levels of MBD3 in different gastric cell lines (GES-1, SGC-7901, HGC-27 and MGC-803). (*P < 0.05, **P < 0.01, ***P < 0.001)

The pan-cancer analysis showed that MBD3 was highly expressed in most types of cancers, including GBM, COAD and STAD (stomach adenocarcinoma, belonging to GC) (Fig. 1A). Due to the limited adjacent normal tissues of gastric cancer in the TCGA database, we combined the data from the GTEx and TCGA databases. We then synthesized the MBD3 expression levels in the dataset. The analysis revealed that MBD3 expression was significantly upregulated in GC tissues compared to normal tissues based on TCGA and GTEx data combined, TCGA separate dataset, and TCGA-paired dataset (Fig. 1B-D). Figure 1E shows IHC sections of GC tissues and adjacent GC tissues from three elderly male patients at 20 × and 40 × magnification, respectively, and representative images indicated that MBD3 expression in GC tissues was higher than that in paracancerous tissues. To investigate whether MBD3 is overexpressed in GC cells, we detected the MBD3 expression in GES-1, SGC-7901, MGC-803 and HGC-27 cells, and found that MBD3 was higher in GC cells than normal gastric epithelial cells and was relatively highest in HGC-27 cells, second highest in MGC-803 cells and lowest in SGC-7901 cells (Fig. 1F, G). The results indicated that MBD3 may serve as an oncogene in GC.

### MBD3 is associated with poor prognosis in GC

To explore the relationship between the MBD3 expression level and prognosis of GC, we analyzed the association between the MBD3 expression level and patient survival based on the TCGA dataset. The risk score map and survivorship curve suggested that the high MBD3 expression group of GCs had a worse prognosis (Figure S1A-C). Then, a nomogram based on independent OS factors was established to integrate MBD3 expression as a GC biomarker via calibration analysis (Figure S1D). The total score ranged from 1 to 100 is the sum of the scores of each variable and higher total points indicated a worse prognosis (Figure S1D). Among them, N3 stage was 80 points, PD&PR&SD was 100 points, and the 1-year survival rate was 70%, which was significantly better than the 2-year and 3-year survival rates. The ideal line and the prediction line of 1, 2, and 3 years fit well, indicating that the nomogram had reasonably accurate prediction efficiency (Figure S1E). As shown in univariate Cox regression models, we analyzed the influence of MBD3 on prognosis in subgroups. The results of the univariate analysis demonstrated that for T3 (adjusted HR = 1.713, 95% CI = 1.103–2.660, P = 0.016), T4 (adjusted HR = 1.729, 95% CI = 1.061–2.819, P = 0.028), N1 (adjusted HR = 1.629, 95% CI = 1.001–2.649, P = 0.049), N3 (adjusted HR = 2.709, 95% CI = 1.669–4.396, P < 0.001), M1 (adjusted HR = 2.254, 95% CI = 1.295–3.924, P = 0.004), CR (adjusted HR = 0.237, 95% CI = 0.163–0.344, P < 0.001), and Age > 65 (adjusted HR = 1.620, 95% CI = 1.154–2.276, P = 0.005) were independent factors of OS in patients with GC (Figure S1F). While the multivariate analysis results showed that N3 stage (adjusted HR = 1.972, 95% CI = 1.087–3.579, P = 0.025), CR (adjusted HR = 0.277, 95% CI = 0.186–0.411, P < 0.001), and age > 65 (adjusted HR = 1.576, 95% CI = 1.067–2.329, P = 0.022) were multivariate factors of OS in patients with GC (Figure S1G). The above results suggested that MBD3 was a potential prognostic factor.

### MBD3 promoted migration in GC cells

**Fig. 2:**
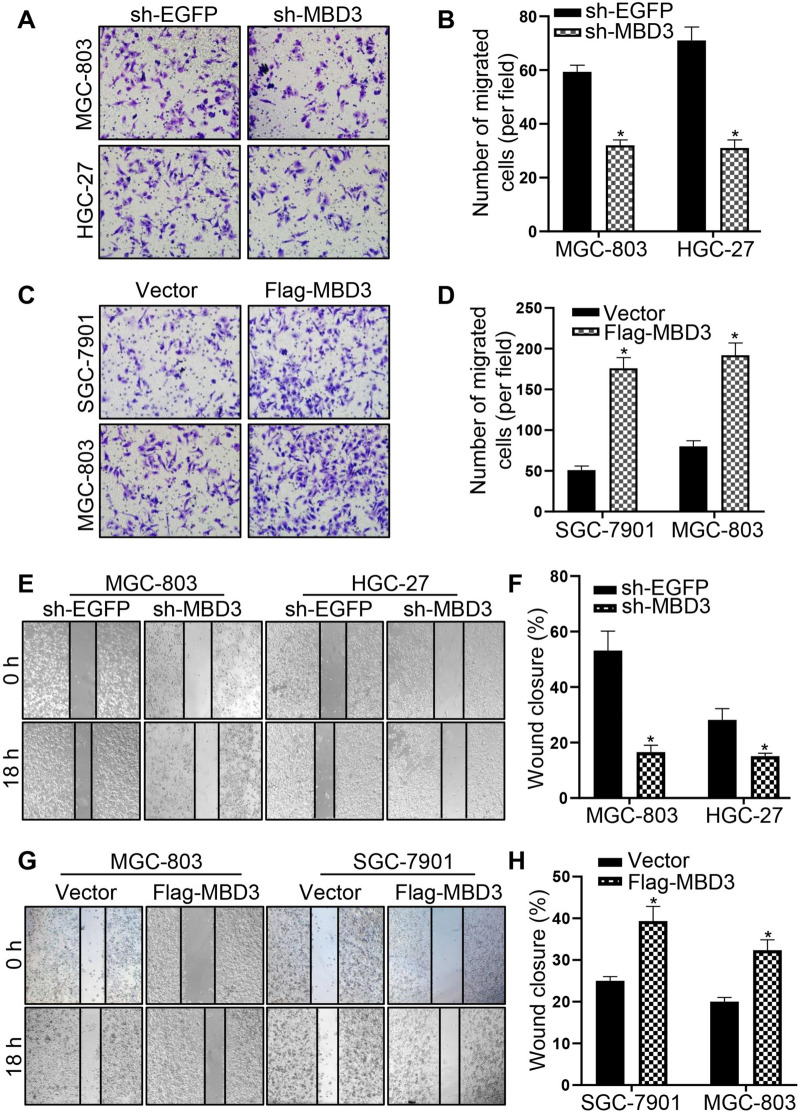
MBD3 enhanced the migration ability of GC cells. **A**, **B** Transwell assays were used to detect the migration ability of MGC-803 and HGC-27 cells transfected with sh-EGFP and sh-MBD3. **C**, **D** Transwell assays were used to examine the migration ability of SGC-7901 and MGC-803 cells transfected with vector and Flag-MBD3. **E** Wound healing assay showed the migration ability of MGC-803 and HGC-27 cells transfected with sh-EGFP and sh-MBD3. **F** Histograms represent the analysis of the wound healing rate in Fig. 2E. **G** Wound healing assay showed the migration ability of SGC-7901 and MGC-803 cells transfected with vector and Flag-MBD3. **H** Histograms represent the analysis of the wound healing rate in Fig. 2H. (*P < 0.05)

Firstly, sh-MBD3 was transfected into MGC-803 and HGC-27 cells with relatively high MBD3 expression while Flag-MBD3 in SGC-7901 and MGC-803 cells with relatively low MBD3 expression, and the results displayed that plasmid transfection was successful (Figure S2A-D). Then, the results of transwell experiment demonstrated that the number of migrated cells was significantly reduced in the sh-MBD3 group of MGC-803 and HGC-27 cells (Fig. 2A, B), whereas many more migrated cells were observed in the Flag-MBD3 group of SGC-7901 and MGC-803 cells (Fig. 2C, D). In addition, the wound healing assay indicated that MBD3 downregulation weakened the migratory capacity of MGC-803 and HGC-27 cells (Fig. 2E, F), and in comparison, MBD3 upregulation increased the motility of SGC-7901 and MGC-803 cells (Fig. 2G, H). The results above showed that MBD3 promoted the migration of GC cells.

### MBD3 enhanced the invasion ability of GC cells

**Fig. 3:**
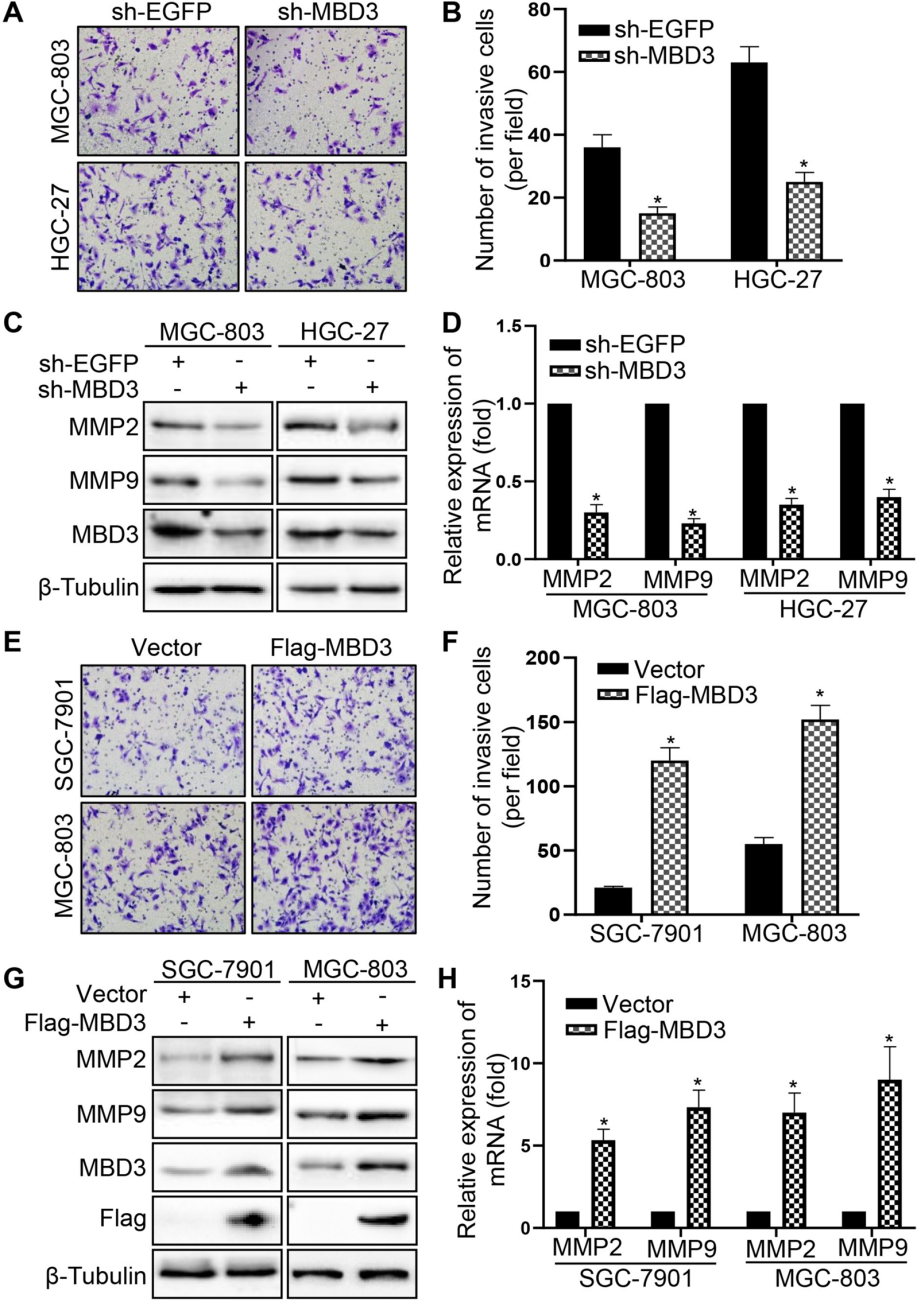
MBD3 enhanced the invasion ability of GC cells. **A**, **B** Transwell invasion assays showed the invasion ability of MGC-803 and HGC-27 cells transfected with sh-EGFP and sh-MBD3. **C**, **D** MMP2 and MMP9 expression were tested by western blot and qRT-PCR in MGC-803 and HGC-27 cells transfected with sh-EGFP and sh-MBD3. **E**, **F** Transwell invasion assays were used to examine the invasion ability of SGC-7901 and MGC-803 cells transfected with vector and Flag-MBD3. **G**, **H** MMP2 and MMP9 expression were tested by western blot and qRT-PCR in SGC-7901 and MGC-803 cells transfected with vector and Flag-MBD3. (*P < 0.05)

Then, we performed a transwell BD assay to detect the invasive capability of GC cells. As shown in Fig. 3A-B, the number of invasive cells transfected with sh-MBD3 decreased in MGC-803 and HGC-27 cells. Besides, MBD3 knockdown decreased invasion associated molecules MMP2 and MMP9 expression in MGC-803 and HGC-27 cells at both the protein and mRNA levels (Fig. 3C, D). In contrast, in SGC-7901 and MGC-803 cells, MBD3 overexpression increased the number of invasive cells (Fig. 3E, F), and reduced MMP2 and MMP9 expression (Fig. 3G, H). The above results demonstrated that MBD3 enhanced the invasive ability of GC cells.

### MBD3 stimulated GC cell proliferation

**Fig. 4:**
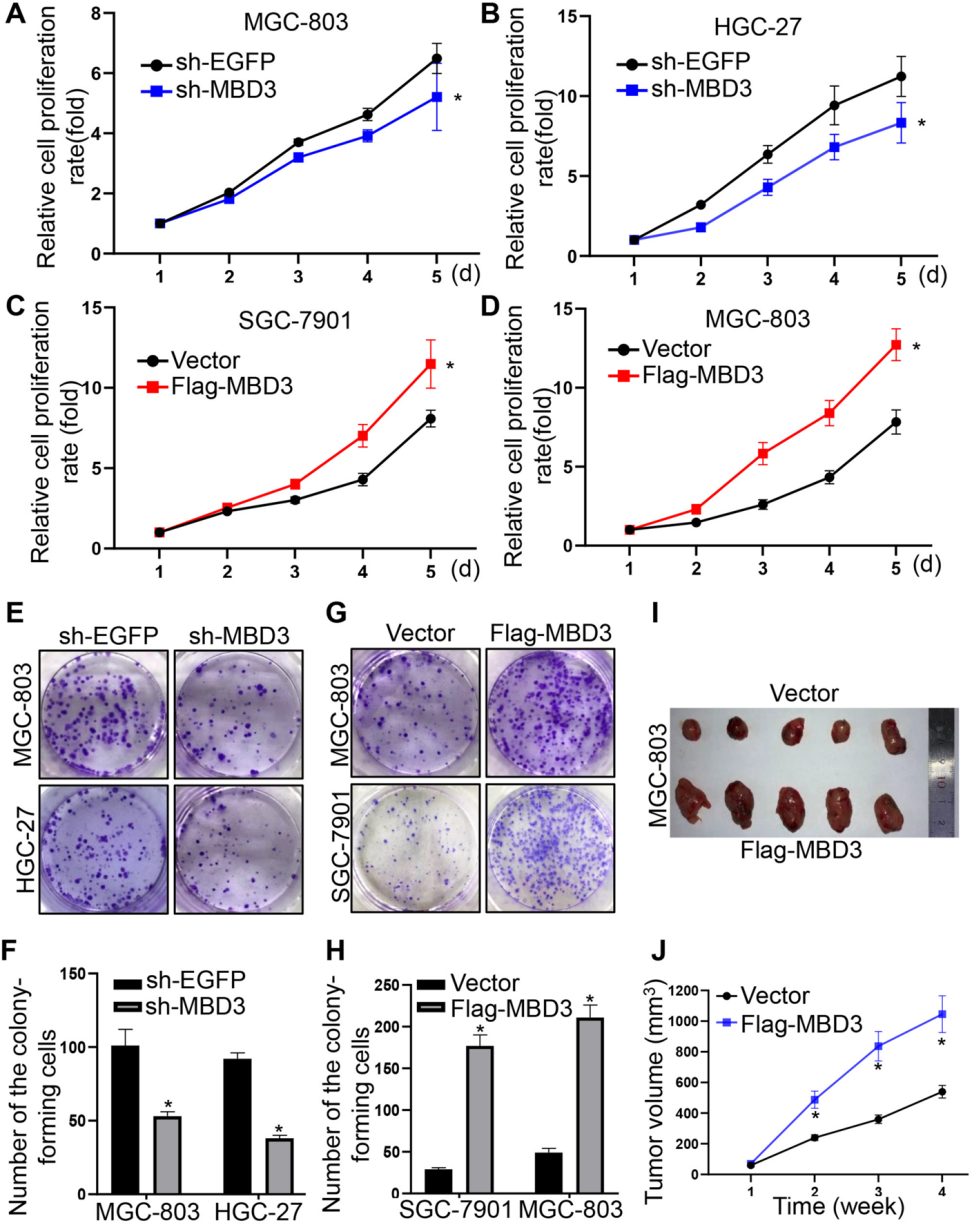
MBD3 promoted GC cell proliferation. **A**, **B** MBD3 downregulation inhibited the proliferation of MGC-803 and HGC-27 cells. **C**, **D** MBD3 upregulation promoted the proliferation of SGC-7901 and MGC-803 cells. All experiments were performed using at least three biological replicates. **E**, **F** MBD3 downregulation weakened colony formation in MGC-803 and HGC-27 cells. **G**, **H** MBD3 upregulation enhanced colony formation in SGC-7901 and MGC-803 cells. **I**, **J** MGC-803 cells with MBD3 upregulation were injected (2 × 10.^6^ cells/site) subcutaneously into a mouse, and the tumor volume was measured weekly (n = 5 mice). (*P < 0.05)

Furthermore, CCK-8 assays showed that MBD3 downregulation slowed the growth of MGC-803 and HGC-27 cells (Fig. 4A, B). In turn, MBD3 upregulation accelerated the growth of SGC-7901 and MGC-803 cells (Fig. 4C, D). In addition, the colony-forming capacity of MGC-803 and HGC-27 cells transfected with sh-MBD3 was decreased (Fig. 4E, F) while MBD3 overexpression enhanced the colony-forming ability of SGC-7901 and MGC-803 cells (Fig. 4G, H). Meanwhile, mice experiments revealed that Flag-MBD3-transfected MGC-803 cells generated larger tumors than control cells (Fig. 4I, J). These results indicated that MBD3 promoted the proliferation of GC cells.

### MBD3 induced EMT via PI3K/AKT pathway activation in GC cells

**Fig. 5:**
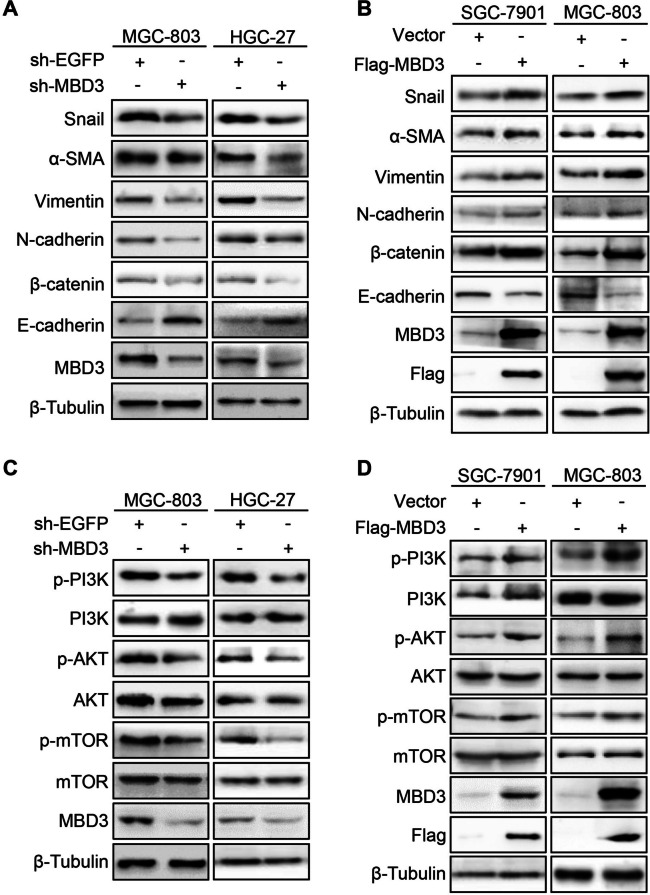
MBD3 promoted EMT in GC cells via activation of the PI3K/AKT pathway. **A** The expression of EMT proteins was examined in sh-MBD3-MGC-803 and HGC-27 cells by western blot. **B** The expression of EMT proteins was examined in Flag-MBD3-SGC-7901 and MGC-803 cells by western blot. **C** Western blotting was used to determine PI3K/AKT pathway protein expression in MGC-803 and HGC-27 cells after transfection with sh-EGFP or sh-MBD3. **D** Western blotting was used to test PI3K/AKT pathway protein expression in SGC-7901 and MGC-803 cells after transfection with vector or Flag-MBD3

The enhanced invasion, migration and proliferation ability was associated with EMT [13]. Therefore, EMT protein markers were examined in GC cells. MBD3 knockdown resulted in E-cadherin upregulation and downregulation of β-catenin, N-cadherin, Vimentin, α-SMA and Snail (Fig. 5A). Meanwhile, MBD3 upregulation contributed to the opposite results (Fig. 5B). As the PI3K/AKT pathway plays a vital role in regulating multiple biological processes such as cell proliferation, cell growth, metabolism and apoptosis [14], western blotting was used to study the relationship between MBD3 and the PI3K/AKT pathway. Compared to the control group, MBD3 downregulation decreased the expression of p-PI3K, p-AKT, and p-mTOR, and the changes in PI3K, AKT, and mTOR expression were not significant in MGC-803 and HGC-27 cells (Fig. 5C), whereas MBD3 overexpression led to the opposite results in SGC-7901 and MGC-803 cells (Fig. 5D). In summary, the results indicated that MBD3 can activate PI3K/AKT signaling to promote EMT in GC cells.

### MBD3 promoted GC cell migration and growth by upregulating ACTG1 expression

**Fig. 6:**
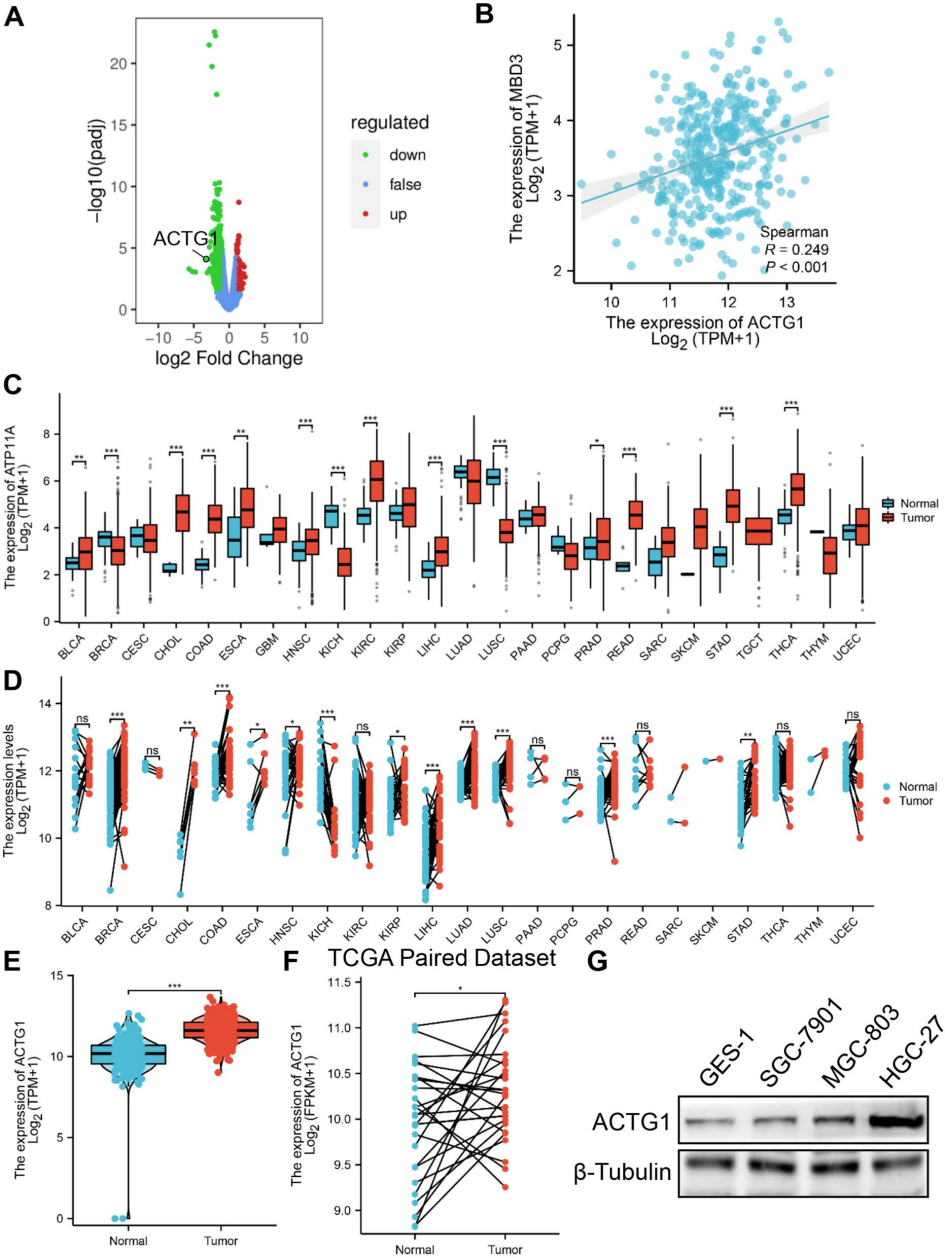
Correlation between MBD3 and ACTG1 and the expression of ACTG1 in GC tissues and cells. **A** Volcano map analysis showed differentially expressed genes in response to sh-EGFP and sh-MBD3 treatment measured by RNA-seq. Green: low expression levels. Red: high expression levels. **B** The relationship between MBD3 and ACTG1 based on the TCGA dataset. **C** ACTG1 expression in different cancer types based on the TCGA database. **D** ACTG1 expression in TCGA-paired cancer and normal tissues. **E** The expression of ACTG1 was higher in GC than in normal gastric tissues from GTEx database (normal 174 patients) and TCGA database (normal 36 patients, tumor 414 patients). **F** ACTG1 expression in TCGA-paired GC and normal tissues (normal 27 patients, tumor 27 patients). **G** Western blotting was used to detect the relative expression levels of ACTG1 in different gastric cell lines (GES-1, SGC-7901, HGC-27 and MGC-803). (*P < 0.05, **P < 0.01, ***P < 0.001)

**Fig. 7:**
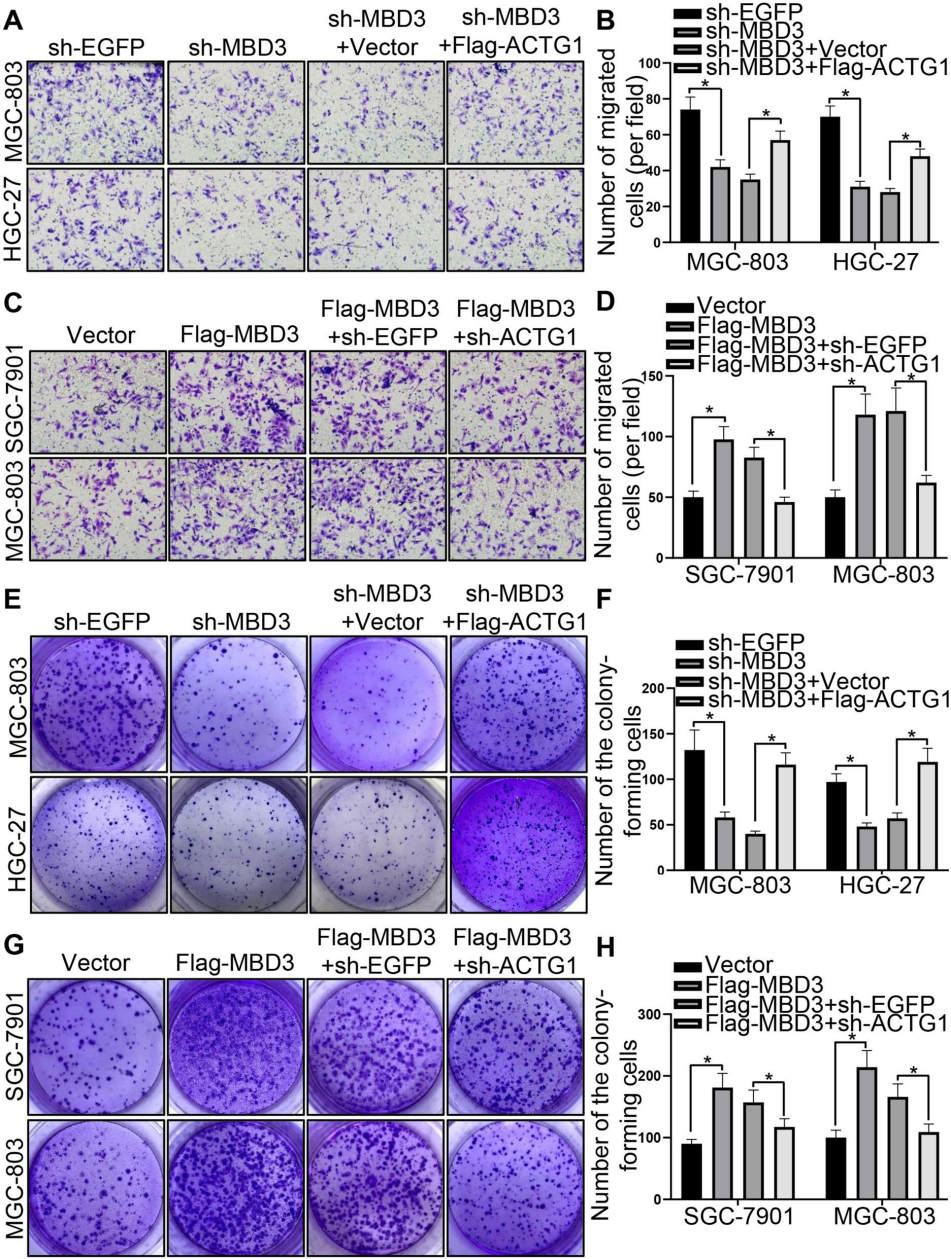
MBD3 promoted GC cell migration and growth by upregulating ACTG1 expression. **A**, **B** The migration ability of MGC-803 and HGC-27 cells transfected with plasmids was tested by transwell assays. **C**, **D** Transwell assays were used to examine the migration ability of SGC-7901 and MGC-803 cells after transfection. **E**, **F** Colony formation was detected in MGC-803 and HGC-27 cells transfected with plasmids. **G**, **H** Colony formation was detected in SGC-7901 and MGC-803 cells after transfection. (*P < 0.05)

To further explore the potential molecular mechanisms, we performed RNA sequencing (RNA-seq) to analyze the gene expression changes in MGC-803 cells transfected with sh-EGFP and sh-MBD3. The statistics of DO enrichment and KEGG classification confirmed that MBD3 participated in GC and the PI3K-AKT pathway (Figure S3A, B). The volcano map analysis of differentially expressed genes revealed that 861 genes were downregulated and 142 genes were upregulated in the sh-MBD3 groups compared with the sh-EGFP group (Fig. 6A). The differentially expressed genes lists are provided in Supplementary Table S1. We focused on the genes related to epithelial polarization, cell migration, and cell–cell junctions for downstream studies, and found that ACTG1 was involved in all of them (Table S1). The mRNA level of ACTG1 decreased in MGC-803 and HGC-27 cells transfected with sh-EGFP and sh-MBD3, while MBD3 overexpression increased the mRNA level of ACTG1 (Figure S2E, F). Then, we confirmed that MBD3 expression was positively correlated with ACTG1 based on the TCGA dataset (Fig. 6B). Subsequently, the role of ACTG1 was explored. The pan-cancer analysis showed that ACTG1 was highly expressed in most types of cancers, especially STAD (Fig. 6C, D). Then, the analysis revealed that ACTG1 expression was significantly upregulated in GC tissues compared to normal tissues based on TCGA separate dataset and TCGA-paired dataset (Fig. 6E, F), and ACTG1 can be a diagnostic and prognostic marker in GC (Figure S4A-F). To verify the RNA-sequencing data, we tested the ACTG1 expression by western blotting. Consistent with the results of RNA sequencing, ACTG1 expression was positively related to MBD3 expression in GES-1, SGC-7901, MGC-803 and HGC-27 cells, higher in GC cells than in gastric normal epithelial cell, and was relatively highest in HGC-27 cells, second highest in MGC-803 cells and lowest in SGC-7901 cells (Fig. 6G). And the ACTG1 plasmids were valid (Figure S5A-E). We selected shACTG1-2 (shACTG1) with the highest knockdown efficiency for further study. The changes of MBD3 in promoting the GC cells migration and proliferation were rescued by the ACTG1 plasmid (Fig. 7A-H). Overall, we demonstrated that MBD3 promoted the malignant progression of GC cells by upregulating ACTG1 expression.

## Discussion

The present study demonstrates that MBD3 expression was higher in GC tissues and cells than in normal tissues and cells. Then, bioinformatic analysis showed that high MBD3 expression was related to poor overall survival in GC. Subsequently, we proved that MBD3 enhanced the migration, invasion and proliferation abilities of GC cells. Furthermore, it was verified that MBD3 activated the PI3K/AKT pathway to upregulate ACTG1 to promote EMT in GC cells.

Cancer cells break the limitation of the tumor microenvironment by changing the genetic and epigenetic landscape [15]. EMT, as a complex reversible process with loss of intercellular cohesion and epithelial cell polarity, is associated with tumorigenesis, metastasis, invasion, and resistance to therapy in cancer [16, 17]. During the process of EMT, cancer cells progress to malignancy and obtain migration ability to invade other areas, with a decrease in epithelial cell adhesion proteins such as E-cadherin and mesenchymal markers such as N-cadherin and Vimentin [18]. EMT has been proven to be one of the main mechanisms for metastasis in GC and is related to poor prognosis [19–21]. For instance, HOXA10 promotes gastric cancer EMT via the TGFB2/Smad/METTL3 signaling axis [22]. Therefore, to promote the development of specific treatment strategies, it is necessary to better understand the mechanism of the EMT process in gastric cancer metastasis.

MBD3, a major member of epigenetics, regulates genes by binding methylated DNA and proteins in various tumors [23–25]. MBD3/NuRD was reported to regulate epithelial-mesenchymal plasticity and tumor metastasis of breast cancer cells [26]. In pancreatic cancer cells, MBD3 inhibits EMT via TGF-β/Smad signaling [9]. Nevertheless, the relationship between MBD3 and EMT in GC is still unclear. In this study, we first analyzed MBD3 expression in the TCGA database and found that MBD3 was overexpressed in GC tissues and cells compared with normal tissues and cells. Then, we demonstrated that MBD3 was associated with poor prognosis in GC. It is implied that MBD3 may serve as an oncogene in GC. Subsequently, we proved that MBD3 enhanced the migration, invasion and proliferation abilities of GC cells. In addition, we provided evidence that MBD3 could induce EMT in GC cells. However, the underlying mechanism is still unclear.

Aberrant activation of the PI3K/AKT/mTOR pathway is involved in several cellular processes in cancers, including metastasis, EMT, autophagy, apoptosis and chemoresistance [27–29]. PI3K is an intracellular phosphatidylinositol kinase, and AKT, as a serine/threonine kinase, is an important downstream target of the PI3K signal transduction pathway [30]. Emerging evidence demonstrates that the PI3K/AKT/mTOR pathway plays a critical role in GC prognosis and metastasis [31]. Therefore, we hypothesized that MBD3 may promote EMT in GC cells through PI3K/AKT pathway. As expected, in our research, we demonstrated that MBD3 significantly activated the PI3K and Akt proteins based on the increased phosphorylation of these proteins. Additionally, we performed RNA-seq analysis to further explore the potential molecular mechanisms. The results of RNA-seq confirmed that MBD3 participated in the PI3K-AKT pathway. Meanwhile, we found that ACTG1 could be the downstream target of MBD3 through the study of genes related to epithelial polarization, cell migration and cell–cell junctions. Subsequently, the analysis and experiments revealed that ACTG1 served as an oncogene in GC and rescued the effect of MBD3 on GC cells. Hence, we suggested that MBD3 upregulated ACTG1 to promote the malignant progression of GC cells via the PI3K/AKT pathway.

In conclusion, our study indicated that MBD3 expression was higher in GC tissues and cells than in normal tissues and cells. MBD3 was correlated with poor prognosis of GC. The data from the present study demonstrated that MBD3 promoted migration, invasion, proliferation and EMT in GC cells. Mechanistically, MBD3 activated the PI3K/AKT pathway to upregulate ACTG1 to promote EMT in GC cells. Our study suggested that MBD3 may serve as a candidate target for GC diagnosis and prognosis.

### Supplementary Information

**Additional file 1:** **Figure S1**. (A) Distribution of the risk score and survival status of MBD3. (B) OS survival curve of MBD3. (C) DSS survival curve of MBD3. (D, E) Prognostic nomogram and calibration analysis of MBD3 at 1, 2 and 3 years. (F) Forest plot of univariate Cox regression analysis. (G) Forest plot of multivariate Cox regression analysis.

**Additional file 2:** **Figure S2**. (A, B) The protein and mRNA levels of MBD3 were tested in sh-MBD3-HGC-27 and sh-MBD3-MGC-803 cells. (C, D) Western blotting and qRT-PCR were used to examine MBD3 expression in MGC-803 and SGC-7901 cells transfected with vector and Flag-MBD3. (E, F) The mRNA level of MBD3 and ACTG1 were detected in GC cells after transfection. (*P < 0.05).

**Additional file 3:** **Figure S3**. (A, B) DO enrichment and KEGG classification were analyzed in MGC-803 cells transfected with sh-EGFP and sh-MBD3 measured by RNA-seq.

**Additional file 4:** **Figure S4**. (A) ROC curve analysis to evaluate the prognostic value of ACTG1 expression in GC examined by TCGA database. (B) The AUC time-dependent curve of ACTG1. (C) Distribution of the risk score and survival status of ACTG1. (D, E) Prognostic nomogram and calibration analysis of ACTG1 at 1-, 3- and 5-years. (F) The prognostic value of ACTG1 expression by univariate analysis.

**Additional file 5:** **Figure S5**. (A) The protein level of ACTG1 and MBD3 were tested in shACTG1-MGC-803 cells after transfection. (B, C) The protein and mRNA levels of ACTG1 were tested in HGC-27 and MGC-803 cells after transfection. (D, E) Western blotting and qRT-PCR were used to examine ACTG1 expression in MGC-803 and SGC-7901 cells transfected with vector and Flag-ACTG1. (*P < 0.05).

**Additional file 6:** **Table S1**. The differentially expressed genes lists based on RNA sequencing analysis in MGC-803 cells transfected with sh-EGFP and sh-MBD3.

## Data Availability

All data generated or analysed during this study are included in this article and its supplementary information files.

## Abbreviations

GC: Gastric cancer
MBD3: Methyl-CpG binding domain protein 3
ACTG1: Actin γ1
EMT: Epithelial-mesenchymal transition
NuRD: Nucleosome remodeling and deacetylase
GTEx: Genotype Tissue Expression Project
TCGA: The Cancer Genome Atlas
IHC: Immunohistochemistry
cDNAs: Complementary DNAs
OS: Overall survival
DSS: Disease specific survival
AUC: The area under the curve
PBS: Phosphate-buffered saline
SD: Standard deviation
ANOVA: Analysis of variance
STAD: Stomach adenocarcinoma
RNA-seq: RNA sequencing
