# Supplementary figures and images for: MBD3 promotes epithelial-mesenchymal transition in gastric cancer cells by upregulating ACTG1 via the PI3K/AKT pathway
Source: Biol Proced Online. 2024 Jan 5;26:1. doi: 10.1186/s12575-023-00228-9 (PMC10768447; doi:10.1186/s12575-023-00228-9)

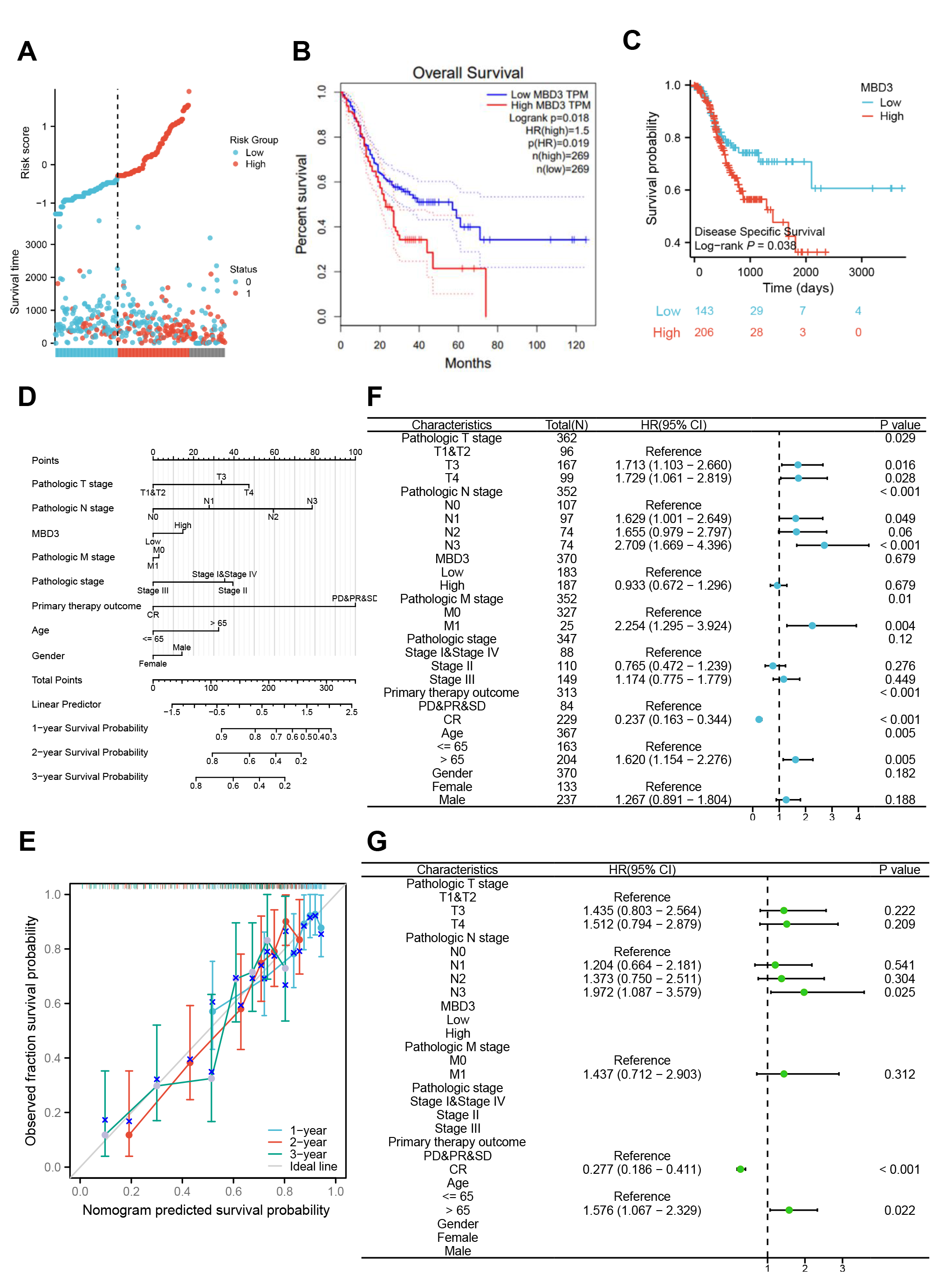

Supplement: Supplementary file 1 — Additional file 1: Figure S1. (A) Distribution of the risk score and survival status of MBD3. (B) OS survival curve of MBD3. (C) DSS survival curve of MBD3. (D, E) Prognostic nomogram and calibration analysis of MBD3 at 1, 2 and 3 years. (F) Forest plot of univariate Cox regression analysis. (G) Forest plot of multivariate Cox regression analysis. [file 12575_2023_228_MOESM1_ESM.tif]

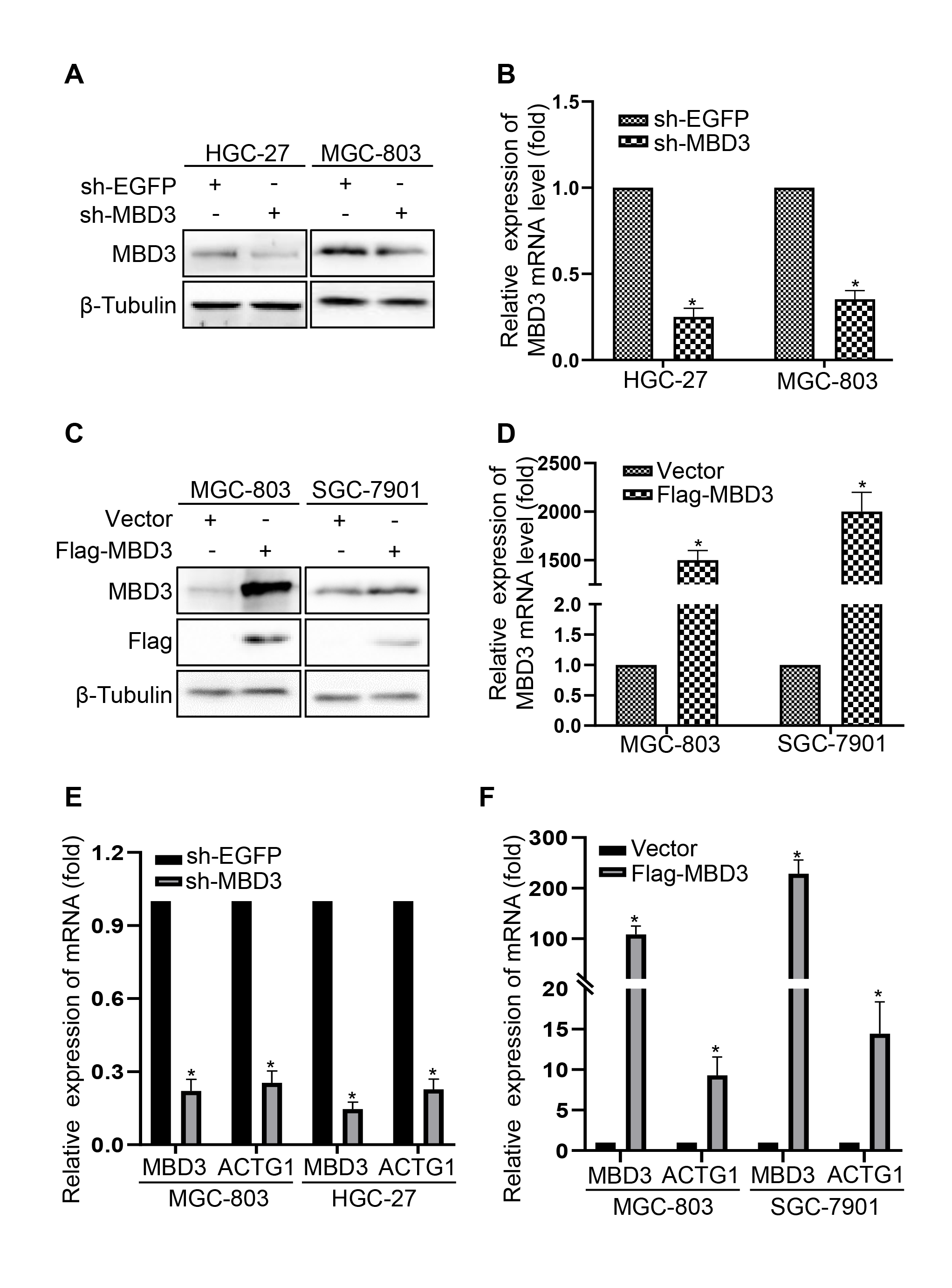

Supplement: Supplementary file 2 — Additional file 2: Figure S2. (A, B) The protein and mRNA levels of MBD3 were tested in sh-MBD3-HGC-27 and sh-MBD3-MGC-803 cells. (C, D) Western blotting and qRT-PCR were used to examine MBD3 expression in MGC-803 and SGC-7901 cells transfected with vector and Flag-MBD3. (E, F) The mRNA level of MBD3 and ACTG1 were detected in GC cells after transfection. (*P < 0.05). [file 12575_2023_228_MOESM2_ESM.tif]

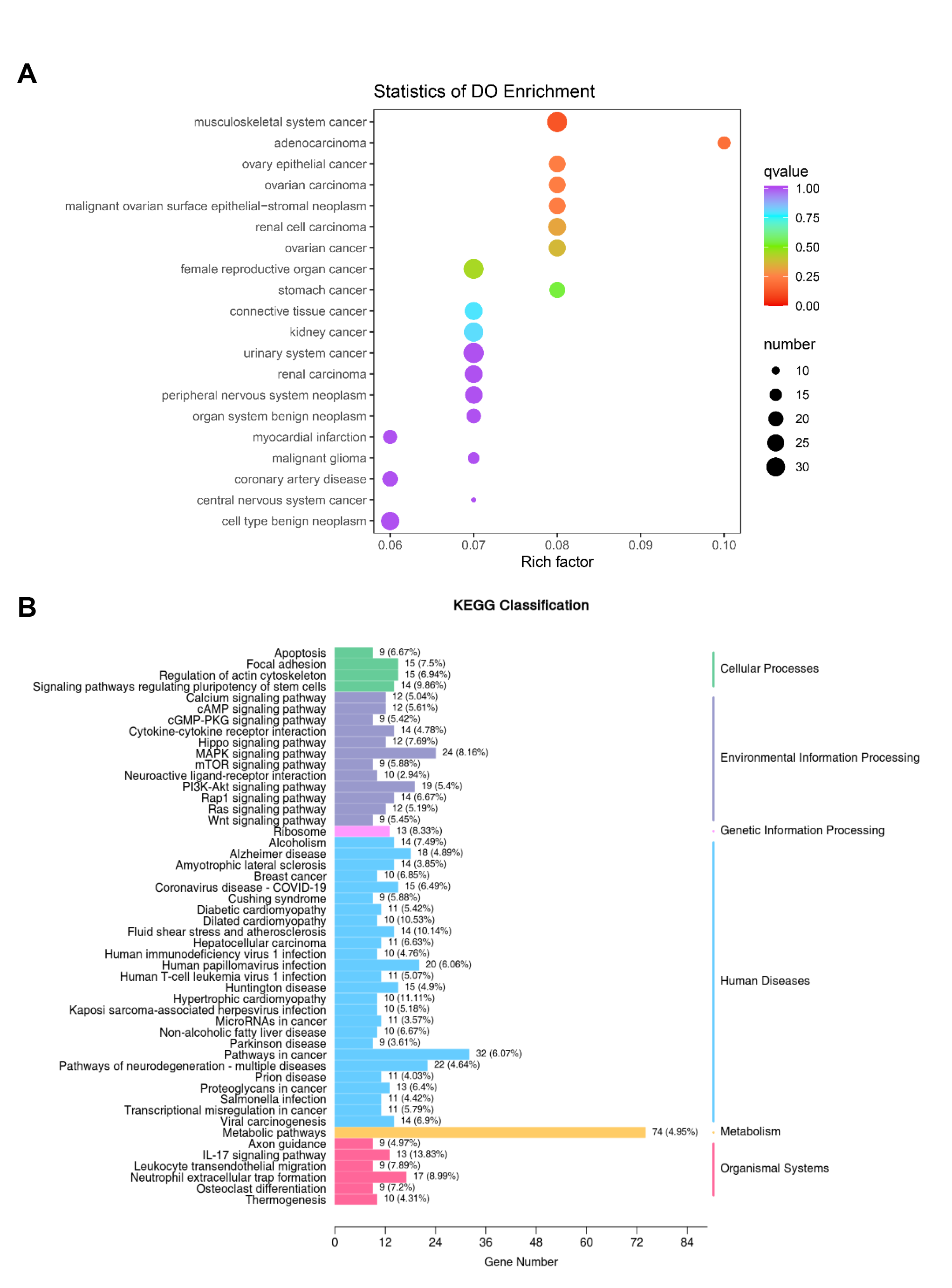

Supplement: Supplementary file 3 — Additional file 3: Figure S3. (A, B) DO enrichment and KEGG classification were analyzed in MGC-803 cells transfected with sh-EGFP and sh-MBD3 measured by RNA-seq. [file 12575_2023_228_MOESM3_ESM.tif]

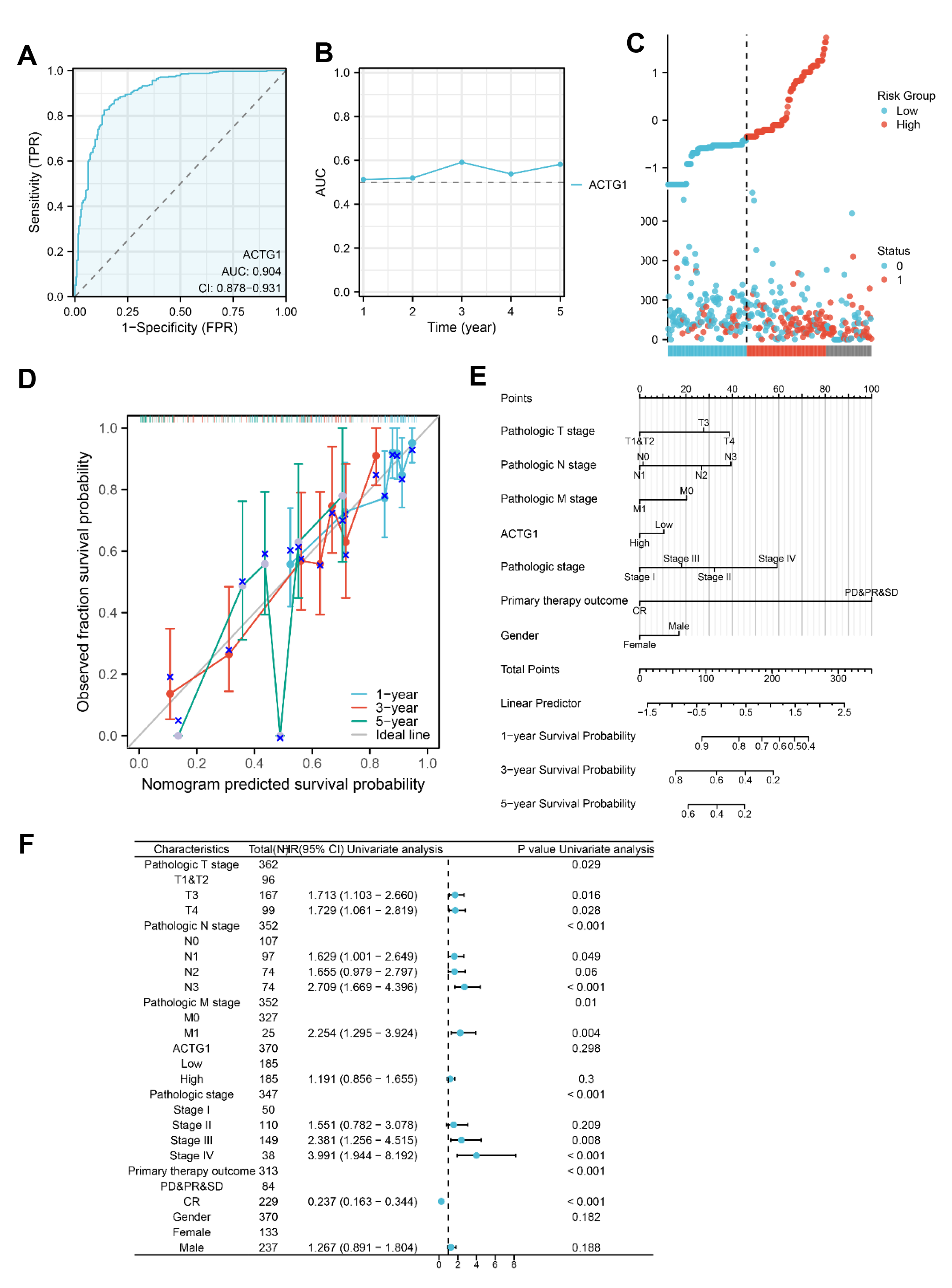

Supplement: Supplementary file 4 — Additional file 4: Figure S4. (A) ROC curve analysis to evaluate the prognostic value of ACTG1 expression in GC examined by TCGA database. (B) The AUC time-dependent curve of ACTG1. (C) Distribution of the risk score and survival status of ACTG1. (D, E) Prognostic nomogram and calibration analysis of ACTG1 at 1-, 3- and 5-years. (F) The prognostic value of ACTG1 expression by univariate analysis. [file 12575_2023_228_MOESM4_ESM.tif]

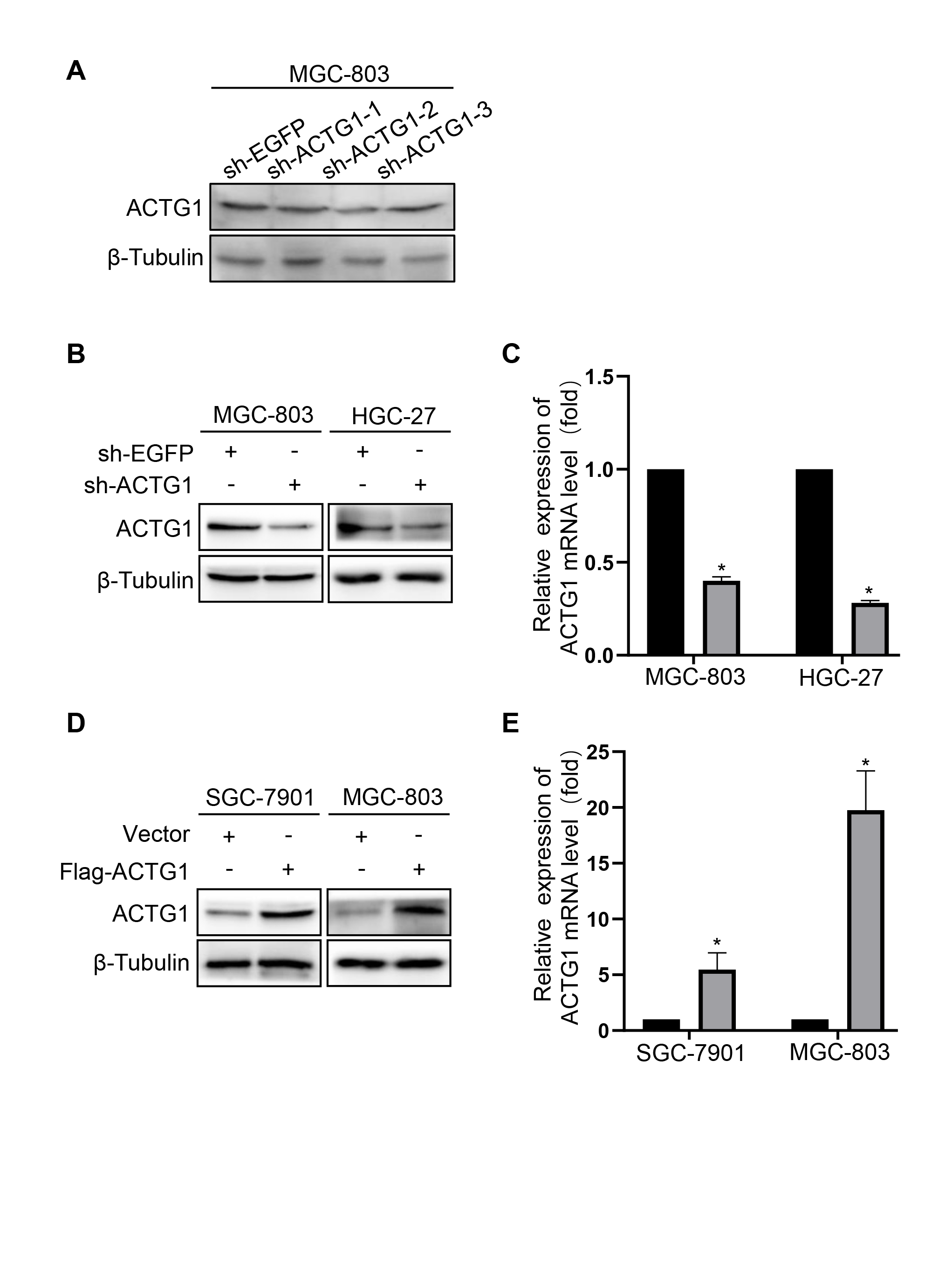

Supplement: Supplementary file 5 — Additional file 5: Figure S5. (A) The protein level of ACTG1 and MBD3 were tested in shACTG1-MGC-803 cells after transfection. (B, C) The protein and mRNA levels of ACTG1 were tested in HGC-27 and MGC-803 cells after transfection. (D, E) Western blotting and qRT-PCR were used to examine ACTG1 expression in MGC-803 and SGC-7901 cells transfected with vector and Flag-ACTG1. (*P < 0.05). [file 12575_2023_228_MOESM5_ESM.tif]
